# Awareness and knowledge of drug decriminalization among people who use drugs in British Columbia: a multi-method pre-implementation study

**DOI:** 10.1186/s12889-024-17845-y

**Published:** 2024-02-08

**Authors:** Alissa Greer, Jessica Xavier, Olivia K. Loewen, Brooke Kinniburgh, Alexis Crabtree

**Affiliations:** 1 School of Criminology, Simon Fraser University, 8888 University Drive, Burnaby, BC V5A1S6 Canada; 2 British Columbia Center for Disease Control, 655 W 12th Ave, Vancouver, BC V5Z 4R4 Canada; 3 Public Health Agency of Canada, Sinclair Centre Federal Building, 218-757 Hastings St. West, Vancouver, BC V6C 1A1 Canada

**Keywords:** Decriminalization, Drug policy, Qualitative, Quantitative

## Abstract

**Background:**

In January 2023, British Columbia implemented a three-year exemption to *Controlled Drugs and Substances Act*, as granted by the federal government of Canada, to decriminalize the personal possession of small amounts of certain illegal drugs. This decriminalization policy, the first in Canada, was announced in response to the overdose emergency in British Columbia as a public health intervention that could help curb overdose deaths by reducing the impact of criminalization and increasing access to health and social services through stigma reduction.

**Methods:**

The current multi-method study examines people who use drugs’ awareness and knowledge of British Columbia’s decriminalization model through cross-sectional quantitative surveys and qualitative interviews among people who use drugs from September–November 2022, immediately prior to the implementation of decriminalization.

**Results:**

Quantitative findings show that two-thirds (63%) of people who use drugs were aware of the policy, but substantial knowledge gaps existed about the legal protections afforded (threshold amount, substances included, drug trafficking, confiscation). The qualitative findings suggest that people who use drugs misunderstood the details of the provincial decriminalization model and often conflated it with regulation. Results suggest that information sharing about decriminalization were minimal pre-implementation, highlighting areas for knowledge dissemination about people who use drugs' rights under this policy.

**Conclusions:**

Given that decriminalization in British Columbia is a new and landmark reform, and that the success of decriminalization and its benefits may be undermined by poor awareness and knowledge of it, efforts to share information, increase understanding, and empower the community, may be required to promote its implementation and benefits for the community.

## Background

In 2022, the Government of British Columbia (BC) announced that it received approval from the Canadian federal government to decriminalize the personal possession of illicit drugs for adults in the province [1]. Drug decriminalization in BC is planned as a three-year trial under an exemption from Sect. 56(1) of Canada’s *Controlled Drugs and Substances Act*, granted by Health Canada [2]. The BC decriminalization model formally eliminates criminal penalties for the possession of drugs under certain parameters. The exemption only applies to adults over the age of 18 in BC and to possession of 2.5 g cumulatively of certain drugs (including MDMA, crack and powder cocaine, methamphetamine, and opioids, such as heroin and fentanyl) for personal use. Possession of substances not mentioned above, including novel psychoactive substances, remains illegal in BC. It is a no-sanction model; alternative penalties, such as fines or mandatory treatment, are not included. However, police officers can provide health and social service information cards to individuals who request them. Under the BC model, the market itself remains illegal; the sale and exchange of any weight of drugs remains a drug trafficking offense under Canada’s *Controlled Drugs and Substances Act*. The exemption also does not apply to certain circumstances, such as at schools, airports, and parks, as well as in vehicles and for individuals with drug-related court conditions. The initial exemption for this decriminalization model will be piloted in BC for three years, from January 31, 2023 to 2026, when internal and external groups will evaluate it [2].

Drug decriminalization in BC follows years of advocacy by public health experts, police officers, people who use drugs (PWUD), and other drug policy advocates to staggering drug toxicity death rates [3–6]. BC, like other parts of Canada and the United States, has been experiencing unprecedented drug-related death rates that are linked to drug toxicity in the unregulated and illicit market [7]. Since a public health emergency was declared by the provincial government in 2016, BC Corners Service has recorded nearly 13,000 drug toxicity deaths [8]. In response to this crisis, a host of policy interventions have been introduced, including the expansion of take-home naloxone and drug treatment, and the establishment of overdose prevention sites, anti-stigma campaigns, and drug checking programs [9, 10]. As well, following the onset of the Covid-19 emergency, a policy was introduced in BC to signal government support for off-label prescribing of pharmaceutical medications as an alternative to the illicit drug market, an initiative known as ‘prescribed safer supply’ [11]. Nationally, BC was also impacted by Canada’s *Good Samaritan Drug Overdose Act* introduced in 2017 that decriminalized personal drug possession at overdose events in an attempt to promote calling for emergency assistance at overdoses [10, 12].

However, as drug toxicity deaths have not waned in recent years, alternative drug policies remained a priority. In their announcement on decriminalization, the Government of BC stated their hopes to address overdose by “…reducing barriers and stigma that prevent people from accessing life-saving supports and services” [13]. Some, including BC’s Chief coroner [14], have critiqued this goal as being too far removed from practical measures to reduce overdose deaths, such as addressing the toxic supply [15, 16]. Nevertheless, the provincial and federal governments intend to reframe drug use from a criminal issue to a health issue to promote greater dialogue about drug use, supports, and not using drugs in isolation [13, 17]. This aim follows research showing that criminal drug laws and policing exacerbate social and health risks, including overdose risk, due to fear of police, social stigma, and isolation risk [18–22].

While introducing decriminalization can be viewed as responsive to the drug toxicity crisis, its impacts are not necessarily automatic. Evidence shows that drug policy objectives can be thwarted by poor implementation efforts and a lack of knowledge among those it is meant for [23–27]. For instance, details on how police officers will assess the content of drugs remains unclear. Therefore, key to the implementation of decriminalization in BC is promoting awareness and knowledge of the policy itself among the groups it is intended for. To promote the uptake and benefits of decriminalization, knowledge sharing among PWUD, police officers, and the public about the existence and details of the policy is required.

The current study aimed to examine the awareness and knowledge of BC’s incoming decriminalization model among PWUD prior to implementation.

### Literature review

In recent years, the prohibition of drugs and criminalization of people who use them have been under a spotlight given the related harms from policing. A large body of evidence suggests that policing is a determinant of health for PWUD, and that police harassment has resulted in negative health outcomes for them. For example, police presence in drug markets results in rushed injections and increased overdose risk [28–33]. Other studies show that policing discourages PWUD from carrying and/or using harm reduction equipment out of fear of arrest and harassment [28, 32]. Both policing and the stigma associated with criminalization and drug use also negatively impacts people accessing health and harm reduction services, including syringe programs, overdose prevention sites, opioid substitution therapy and HIV medication [34–38]. Given these harms, researchers suggest that policing PWUD has contributed to the ‘global epidemic’ of HIV and hepatitis C [20].

A growing body of research also suggests that criminalization and policing is related to increased overdose risk. Fear of arrest is a barrier to people seeking emergency medical services or calling 9–1-1 during overdose events [39–43]. As well, the stigma associated with drug use, a structural consequence of criminal drug laws [44], is a main factor keeping people silent about their drug use, causes people to use drugs alone, and limits people’s access to overdose prevention services [45–47]. Specific police practices are also associated with greater overdose risk. For instance, in a recent retrospective cohort study of administrative data following drug market disruption attempts by policing, researchers found that increased drug confiscations by police officers were significantly associated with a two-fold increase in fatal overdoses in surrounding neighborhoods in the three weeks following enforcement [21].

In response to the overdose crisis and evidence of the impact of criminalization on overdose, several jurisdictions, including in Canada, passed drug-related good Samaritan laws, which decriminalizes personal drug possession at the scene of an overdose. However, recent studies show limited effectiveness of such policies on willingness to call 9–1-1 and overdose risk due to a lack of knowledge and awareness of the policy itself [25, 27, 41, 48], although there are differences in the populations who are knowledgeable on this Act [49]. Similar results are found in other jurisdictions, including several states in the United States with drug-related good Samaritan laws [26, 39, 50–52]. Subsequently, PWUD express ongoing fear of arrest and police intervention at overdose events, undermining drug-related good Samaritan laws achieving desired benefits.

In other decriminalized jurisdictions such as Portugal and Oregon, there is only a small body of literature on the impact and implementation of reforms, although some research points to the importance of knowledge and awareness of reforms among both people impacted by decriminalization (i.e., PWUD) and those responsible for taking it up in practice (i.e., police officers and other administrators) [53]. For example, in Mexico, a mixed-methods study found that only 11% of over 700 PWUD were aware of drug decriminalization two years after its implementation, and recent police encounters were still associated with syringe sharing [53]. This study and others also suggest that lack of knowledge and awareness of decriminalization can undermine its benefits or impact [23–27]. To our knowledge, however, no studies have been conducted on knowledge and awareness of drug decriminalization prior to its implementation in any jurisdiction with such a reform.

In the current study, we present qualitative and quantitative findings relating to awareness and understanding of specific details about BC’s decriminalization model. Data collection took place between September 2022 and January 2023, immediately after the exemption was announced (May 31, 2022) and before its inception (January 31, 2023). Quantitative and qualitative data on PWUDs’ awareness and understanding of BC’s decriminalization policy during this time provides a robust baseline of PWUD’s knowledge of the legal change, including identifying gaps and lessons learned for future efforts in BC, Canada, and beyond.

## Methods

In this study, we draw on two different sources of data – one quantitative and one qualitative – that were collected concurrently but independently. Data collection for both data sources took place between September 2022 and January 2023, immediately prior to the implementation of decriminalization, across the province of BC.

While data were collected separately and independently, each research team, qualitative and quantitative, informed both data collection instruments to ensure corroboration of data across methods and findings. We ensured qualitative interview questions could provide greater understanding of PWUDs’ knowledge and awareness of decriminalization beyond the presence and prevalence of these factors obtained from the survey data. For instance, PWUDs’ descriptions about how they became aware of decriminalization pointed to the nature of the discourse and information exchange in the community. Conversely, the survey provided an opportunity to understand predictors of knowledge and awareness, such as regional and socioeconomic differences, which pointed to areas of further examination in the qualitative data. The interview guide revisions also included asking about public drug consumption in a way that complemented the survey questions.

The current study leverages the complementary natures of these two data sources. To triangulate findings, the investigators met to compare, examine, and discuss the findings on outcomes related to awareness and knowledge of decriminalization in BC. Following data collection and preliminary analysis, the investigators met to triangulate findings by examining and comparing findings on outcomes related to awareness and knowledge of decriminalization in BC. Findings from both the quantitative and qualitative data are grouped under four topic domains: (1) awareness of decriminalization; (2) understanding of decriminalization; (3) knowledge of decriminalization; and (4) sources of information about decriminalization. For each topic domain below, we provide a description of the survey and interview findings, considering how the qualitative can complement and help explain the quantitative findings. Prior to data collection, both the quantitative and qualitative data teams connected independently with a community advisory board, the BC Center for Disease Control’s Professionals for the Ethical Engagement of Peers (PEEP). This group was comprised of six people with past or present experience of illegal drug use who were well-connected with other networks and groups of PWUD across BC. Members were compensated for their involvement at a rate that aligned with local standards. For data collection, the group advised on study recruitment and sampling strategy, and the questions asked in the data collection instruments. Members also assisted in participant recruitment for the qualitative study by sharing the study flier, helping with scheduling interviews, and distributing the consent form and honorarium to participants.

After the data were analyzed, the study findings were presented back to the group for additional feedback, discussion, and validation.

### Quantitative methods

*Sampling, Recruitment, and Data Collection:* Quantitative data was collected through the Harm Reduction Client Survey which is a long-running survey of clients at harm reduction supply distribution sites across BC. Data collection for this survey leverages the centralized Provincial Harm Reduction Supply Program run by the BC Center for Disease Control. The survey supports rapid information gathering on the health of PWUD and for quality improvement of the Provincial Harm Reduction Supply Program. Methods for the survey have previously been described in depth [54].

In brief, harm reduction distribution sites are sampled annually from diverse sizes of communities across BC. At participating sites, a paper-based survey instrument was used to collect data. Data collection was supported by site staff or peer workers who explained the survey to clients, offered the opportunity to participate, and obtained verbal informed consent while reassuring individuals that participation was voluntary and anonymous. Inclusion criteria were being at least 19 years of age and reporting use of a drug that is illegal, opioid agonist therapy, or prescribed safer supply in the previous six months. Participants were recruited from 29 harm reduction supply sites located in a range of large, medium, and small communities across BC. Data collection occurred between November 2022 and mid-January 2023.

The Harm Reduction Client Survey addresses a variety of other topics, including harm reduction service needs, in keeping with its purpose as a surveillance and quality improvement initiative of the Provincial Harm Reduction Supply Program. Questions about decriminalization were added to the survey in 2022 following the announcement that the BC government would be implementing this policy in 2023 [55]. Survey questions specifically about decriminalization assessed: (a) knowledge and awareness; (b) drug purchasing patterns; (c) experiences with police and health services; (d) barriers to accessing supportive services. We also included questions on demographic characteristics, substance use patterns, internet access, and housing concerns. The quantitative study was granted ethics approval from the University of British Columbia (H07-00570).

### Data management and analysis

To support consistency and quality, clear guidelines were developed and shared with staff engaged before data entry began, and small modifications were made and communicated to account for variations in question completion. Responses were entered into a REDCap database and extracted after data entry completion. Data cleaning included grouping write-in responses with existing categories and creation of new categories if necessary.

The analytic sample was selected based on the outcome variable and complete responses to the predictor variables. Participants who answered “yes” or “no” to the question “*Did you know that BC has a new decriminalization policy starting January 31, 2023? Under this policy, it is not a crime to possess small amounts of some illegal drugs for personal use”* were included. Those who answered “yes” were classified as the outcome group and individuals who answered “no” classified as the comparison group. Of the 503 participants with valid responses to the survey, 404 (80%) had complete information and were included in the analysis. There were no significant differences in the sociodemographic factors between the full and analytic sample.

Among participants aware of decriminalization, we examined knowledge of decriminalization through responses to two true or false questions about the details of decriminalization: “*Police can confiscate/take away drugs if you are holding less than the allowable amount”;* and *“People can be arrested for drug trafficking/dealing, no matter how much drug they have on them”*. Responses to each question were scored as correct (1) vs incorrect or not sure (0). To assess a greater understanding of decriminalization, participants were scored on getting both questions correct (1) vs one or zero correct (0). Knowledge of the specific substances included under decriminalization were examined with participants responding “yes” or “no” to being aware of each of the five substances.

Table 1 summarizes the demographic characteristics of the sample, also considered as predictor variables. Gender identities were cis man, cis woman, and gender expansive or transgender, which included gender non-conforming, trans-man, trans-woman, or other identities. Due to small counts, the latter category was not included in the multivariate analysis. Participants reported their age in years, which was categorized into 19–39 years and 40 years or older. Community size was derived from the harm reduction site at which the survey was completed, and categorized into large (> 100,000), medium (30,000 to 99,999), and small (1,000 to 29,999) population centres. BC has five regional health authorities that deliver health services (Fraser, Interior, Island, Northern, and Vancouver Coastal); the health authority the survey was completed in was used to categorize participants. Concern about losing housing in the last 6 months, internet access, opioid use in the last three days, and stimulant use in the last three days were all as categorized yes or no according to survey responses.

**Table 1:** Quantitative sample characteristics, self-reported (*n* = 404)

	***N***	**%**
**HA of survey**		
Interior	105	26.0%
Fraser	85	21.0%
Vancouver Coastal	40	9.9%
Island	91	22.5%
Northern	83	20.5%
**Community size**		
Large urban centre	143	35.4%
Medium population centre	120	29.7%
Small population centre	141	34.9%
**Type of current residence**		
Private or band-owned residence, alone or with others	101	25.0%
Another residence (e.g., hotel/motel, SRO, supportive housing)	99	24.5%
Shelter	78	19.3%
No regular place to stay (homeless, tent, couch-surf)	112	27.7%
Unknown / Did not answer	14	3.5%
**Concerned about losing housing in the last 6 months**		
Yes	240	54.0%
No	164	39.0%
**Have a cell phone**		
Yes, with or without minutes or a plan	216	53.5%
No	186	46.0%
Unknown / Did not answer	2	0.5%
**Internet Access**		
Yes	334	82.7%
No	70	17.3%
**Age group, in years**		
19–29	59	14.6%
30–39	127	31.4%
40–49	110	27.2%
50 or older	108	26.7%
**Sex/Gender**		
Cis woman	149	37.0%
Cis man	255	63.0%
**Ethnicity**		
Indigenous, alone or in combination	191	47.3%
White only	191	47.3%
Other racialized identities	6	1.5%
Unknown / Did not answer	16	4.0%
**Sexual orientation**		
Heterosexual or straight	341	84.4%
Lesbian or gay	9	2.2%
Bisexual or pansexual	30	7.4%
Asexual	3	0.7%
Unsure/questioning	2	0.5%
Unknown / Did not answer	19	4.7%
**Employment**		
Full-time (≥ 30 h/week)	69	17.1%
Part-time (< 30 h/week)	18	4.5%
No	308	76.2%
Unknown / Did not answer	9	2.2%
**Frequency of substance use in the last 30 days**		
Daily	271	67.1%
A few times/week	68	16.8%
A few times/month	31	7.7%
Did not use	18	4.5%
Unknown / Did not answer	16	4.0%
**Injection drug use, last 6 months**		
Yes	154	38.1%
No	243	60.1%
Unknown / Did not answer	7	1.7%
**Inhalation drug use, last 6 months**		
Yes	347	85.9%
No	47	11.6%
Unknown / Did not answer	10	2.5%
**Drug use at overdose prevention or supervised consumption site, past 6 months**		
Used OPS/SCS	191	47.3%
Did not use OPS/SCS	207	51.2%
Unknown / Did not answer	6	1.5%

All quantitative analyses were conducted in R (version 4.2.1). To assess predictors of a person being aware of decriminalization a multivariate log binomial model was fitted with participant characteristics. A log binomial model was chosen as awareness of decriminalization is binomial outcome that is not rare. Model output is presented as Risk Ratios (RR), wherein RR > 1 indicates an increased association with being aware of decriminalization in the exposure group compared to the reference group with statistical significance defined by the 95% confidence internal not crossing the null. Bivariable analyses compared levels of each predictor between the outcome and comparison groups using Chi-squared tests of independence with statistical significance defined at *P* < *0.05.* To assess predictors of a participant correctly answering each true or false question and getting both questions correct, three multivariate log binomial models were fitted with participant characteristics. For each knowledge outcome, bivariable analyses compared levels of predictors between the outcome and comparison groups using Chi-squared tests of independence with a statistical significance defined at *P* < *0.05.* For each of the five drugs included in decriminalization, we examined if people used the drug were more likely to be aware of its inclusion in decriminalization. Use of the drug in the last 30 days was treated as the exposure and awareness of its inclusion as the outcome in a Chi-squared test of independence with a statistical significance defined at *P* < *0.05.*

### Qualitative methods

*Sampling, Recruitment, and Data Collection:* For the qualitative interviews, PWUD were recruited using targeted and snowball sampling approaches. To recruit interview participants, CAB members and peer workers distributed recruitment fliers through their networks of PWUD that spanned the same regions sampled for the Harm Reduction Client Survey. These individuals also facilitated scheduling and honorarium payment. In some cases, provided telephones for the interview itself. To reduce bias and gain diversity in perspectives, we also purposefully sampled certain groups to ensure diverse perspectives were captured, such as unhoused, racialized, and gender diverse individuals. Recruitment and data collection continued until the research team felt they had met saturation for the aims of the study (*n* = 38), which occurred in early January 2023. The qualitative sample characteristics are in Table 2.

**Table 2:** Qualitative Sample Characteristics, self-reported (*N* = 38)

	***n***	**%**
**Race/Ethnicity**		
White	18	47.4%
Indigenous	17	44.7%
Racial minority	3	7.9%
**Gender**		
Cisgender Man	19	50.0%
Cisgender Woman	18	47.4%
Gender expansive/non-binary	1	2.6%
Two Spirt	1	2.6%
**Living circumstances**		
Private residence	13	34.2%
Homeless/No regular place to stay	7	18.4%
Other residence (shelter, SRO)	18	47.4%
**Region**		
Vancouver Coastal	10	26.3%
Fraser	6	15.8%
Northern	8	21.1%
Island	7	18.4%
Interior	7	18.4%
**Drugs used**^**a**^		
Polysubstance use	36	94.7%
Opioid	23	60.5%
Stimulant	33	86.8%
Other (e.g. benzos)	24	63.2%
**Age (in years)**		
31–58 (mean = 40)		

^a^Participants could indicate more than one drug used

Inclusion criteria were: (a) 19 years or older, (b) self-identify as someone who has used illegal drugs (e.g., cocaine, opioids, methamphetamine) in the past six months, (c) available via telephone or Zoom, (d) interested in discussing decriminalization and recent police interactions.

Prior to the interview, participants provided informed consent. Eligible and interested participants were provided with a consent form that explained the purpose of the study and the requirements of participation. Verbally and through the consent form participants were informed that their participation was voluntary and confidential. The consent process and interviews were conducted by a trained research assistant or coordinator.

All interviews were conducted over the phone or in person and digitically audio recorded following informed consent. We conducted a total of 38 qualitative interviews – a sample size that was guided by informational saturation, meaning that the data were rich enough to produce meaningful patterns and findings [56]. Initially, we felt that we obtained a sense of richness in the data at 32 interviews, but conducted an additonal six to ensure fullness in the dataset and confidence that we achieved a high degree of depth and richness in the data pertaining to the study aims.

The interivews were directed by a question guide organized around three main topics: (a) personal experiences with police officers around drugs, (b) awareness and knowledge of decriminalization, (c) relationship between police and PWUD. The questions asked were developed in collaboration with the research team, community advisory group, and other collaborators and researchers to promote relevance and validity. As well, our team held regular meetings to discuss the quality and relevance of data. From these meetings, we revised some questions to ensure we generated a depth in responses, by adding probing questions, and breadth of information, by adding new topics, that were relevant to our study aims. For example, we added probing questions (“*what benefits do you think there are with decriminalization?”*) and new questions about highly relevant topics introduced previous interviews (“*How do you think police presence in your community will change, if at all, following decriminalization?”*). Revised topics in the question guide received ethical approval before proceeding with additional data collection. A series of demographic questions were also asked consistently to each participant to track the sample and inform the sampling strategy, including age, gender, sex, ethnic/racial identity, living situation, time living in BC, drug type used, and the frequency of harm reduction service use.

### Data management and analysis

After each interview was completed, the audio recording was transcribed verbatim and verified by multiple researchers. Any personally identifying information was removed or anonymized during the transcription process. The anonymization process included removing participant identifiers from data, including names, ages, ethnicity, as well as any identifying experiences, such as details of events. The de-identified transcripts were uploaded to NVivo (a qualitative data coding organization software) to be organized and analyzed [57]. Bi-weekly research team meetings were held to develop a coding framework and included a member of the community advisory board who expressed interest in being involved in the coding and analysis process. As noted, the data were anonymized prior to the community advisory board members reviewing data or findings and was listed on ethics applications and completed and followed Canada’s Tri-Council Policy Statement.

A preliminary framework was applied to five transcripts to ensure that participant perspectives were encapsulated by the codes, and revisions to the framework were made when necessary. Then, the coding framework was applied to all transcripts. Regular meetings were held amongst coders to ensure consistency and reliability and any necessary revisions to the framework and coding were made.

To analyze the data, we engaged in a qualitative descriptive approach guided by the study aims [58]. Qualitative descriptive approaches are often undertaken in mixed- and multi-methods studies, such as ours, to provide descriptions of experiences and perceptions that corroborate and expand quantitative findings – thus making findings overall more meaningful [59]. We looked specifically for indications of participants’ awareness of decriminalization, their understanding of it as a policy framework, and their knowledge of the policy details, regardless of if they were accurate. The analysis process continued into writing and comparing the quantitative results with the qualitative findings to provide a better understanding of knowledge gaps and informational needs of PWUD.

Ethics approval for the qualitative study was granted by Simon Fraser University’s Office of Research Ethics (30-001-251).

## Results

### Awareness of decriminalization: Were PWUD aware of decriminalization?

Two-thirds (63%) of survey respondents were aware of decriminalization at the time of data collection; however, we found significant variations in awareness depending on participants' demographic characteristics (Table 3). Among the strata investigated, awareness of decriminalization was highest among participants: located in Vancouver Coastal Health (75%) and medium population centres (71%); who feared losing their shelter (68%); had internet access (66%); aged 40 years or older (64%); and that identified as cis man (76%). Awareness of decriminalization was the lowest among participants from sites in Northern Health authority (52%), who did not have internet access (50%), or who were 19–39 years old (61%).

**Table 3:** Awareness of BC’s decriminalization policy among harm reduction clients (*n* = 404)

	**Overall (*n* = 404)**		**Aware (*n* = 254)**		**Not aware (*n* = 150)**			**Risk Ratio**	**95% Confidence Interval**		***P*-value**	**χ**^**2**^_**1**_**,*P*-value**
			**n**	**%**		**n**	**%**		Lower CI	Upper CI		
**Health Authority/region**												7.56, 0.11
Fraser	85		53	62.4%		32	37.6%	Ref	Ref	Ref	Ref	
Interior	105		70	66.7%		35	33.3%	1.03	0.85	1.26	0.75	
Island	91		58	63.7%		33	36.3%	1.09	0.91	1.30	0.35	
Northern	83		43	51.8%		40	48.2%	0.93	0.70	1.23	0.60	
Vancouver Coastal	40		30	75.0%		10	25.0%	1.25	1.03	1.51	0.021	
**Community size**												5.65, 0.06
Large population centre	143		81	56.6%		62	43.4%	Ref	Ref	Ref	Ref	
Medium population centre	120		85	70.8%		35	29.2%	1.21	1.01	1.43	0.03	
Small population centre	141		88	62.4%		53	37.6%	1.05	0.89	1.24	0.55	
**Concerned about losing housing in the past 6 months**												5.43, 0.02
No	164		92	56.1%		74	45.1%	Ref	Ref	Ref	Ref	
Yes	240		162	67.5%		78	32.5%	1.21	1.05	1.39	0.01	
**Internet Access**												6.01, 0.01
No	70		35	50.0%		35	50.0%	Ref	Ref	Ref	Ref	
Yes	334		219	65.6%		115	34.4%	1.26	0.99	1.59	0.06	
**Age group**												0.37, 0.54
40 and older	218		140	64.2%		78	35.8%	Ref	Ref	Ref	Ref	
19–39	186		114	61.3%		72	38.7%	1.00	0.98	1.02	0.99	
**Sex/gender**												4.27, 0.04
Cis man	225		170	75.6%		85	37.8%	Ref	Ref	Ref	Ref	
Cis woman	149		84	56.4%		65	43.6%	0.87	0.75	1.01	0.07	
**Opioid used in the last three days**												1.22, 0.27
No		165	109	66.1%		56	33.9%	Ref	Ref	Ref	Ref	
Yes		239	145	60.7%		94	39.3%	0.96	0.85	1.09	0.53	
**Simulant used in the last three days**												4.28, 0.04
No		136	95	69.9%		41	30.1%	Ref	Ref	Ref	Ref	
Yes		268	159	59.3%		109	40.7%	0.82	0.72	0.94	0.004	

^1^Pearson’s Chi-squared test statistic and *p*-value

In the multivariate analysis (Table 3), we found significant differences in awareness of decriminalization based on participant’s access to the internet, concern about losing housing, community size, and health authority. Individuals with access to the internet, those who were concerned about losing housing, and those who participated at sites in Vancouver Coastal were significantly more likely to be aware of decriminalization. Individuals from medium sized communities were 21% more likely to be aware of decriminalization than those from large communities (RR: 1.21 [95% confidence interval (CI): 1.01–1.43]). People who used stimulants in the last 3 days were 18% less likely to be aware of decriminalization than those that did not report using stimulants (RR: 0.82 [95% CI: 0.72–0.94]).

In the qualitative interviews, most had heard of decriminalization in BC; but variations in the depth of knowledge were also found; qualitative analysis revealed possible reasons for these variations. It was evident that some PWUD who were actively involved in decriminalization reforms and advocacy were especially knowledgeable, whereas a few had never heard of it. Although many participants themselves heard about decriminalization, as reflected in the quantitative findings,, many commented on a lack of awareness or conversations about it in their social circles: *“Nobody really even knows about decriminalization.”* (PWUD-01–02); *“nobody’s really talked about it.”* (PWUD-01–35); “*I don’t know. I haven’t really spoken honestly to anybody about it. I didn’t—I don’t know a lot about it myself to be honest with you.”* (PWUD-01–32). For participants, a lack of knowledge amongst PWUD in their social circles was linked to the view that PWUD did not talk about drug policy reforms in their community; for them, discourse and information exchange about decriminalization was minimal.

Some participants indicated that discussions of drug policy changes, including decriminalization, was not necessarily a top priority or topic for PWUD. One person said:

*“But people aren’t even aware of what is going on anyway, nobody really even knows about decriminalization, there should be more public too, like. Nobody – I knew about this thing right when it happened, but nobody else that I told or asked about it – she didn’t know about it and she’s like what… People don’t really know shit… they just go with the program… unless you have an ear, and yourself informed, you will not be informed.”* (PWUD-01–02).  

No participants stated that they learned about the exemption from government-provided materials or campaigns about the incoming decriminalization policy. The combination of limited discourse with minimal information about drug policy reforms, PWUD expressed a burden of responsibility to seek out and acquire such information.

### Understanding decriminalization: do people know what decriminalization is? 

Due to the format of the survey, we did not broadly assess PWUD’s general understanding of decriminalization, such as defining or explaining decriminalization or describing differences from other legal frameworks. However, PWUDs’ understanding of decriminalization was evident in the qualitative interviews where issues arose related to the notion of decriminalization. Although some PWUD were aware of decriminalization, some were quite forward about their lack of understanding: “*No, I have no clue what’s my rights*.” (PWUD-01–07); “*I just didn’t understand it [decriminalization*]” (PWUD-01–09); *“I’ve heard about the word ‘decriminalization’ lots, but I never really got informed of what it was”* (PWUD-01–35). The idea of decriminalization itself was obscure and many were unsure of some or all its features. One feature identified by several participants was the threshold quantity whereas other features, such as possession and trafficking, were unclear:

*“I would figure if you had 2.5 grams or less that they couldn’t take it away. That they would have to give it back to you. And maybe tell you to move somewhere else if you’re using in public or something like that. If you’re by a school or something like that. Maybe…. that’s all I know about the decriminalization. I don’t know too much about it - if they’re allowed to keep the drugs still or what.”* (PWUD-01-07)

The extent to which PWUD understood decriminalization was also articulated through questions they posed to the interviewer. Some asked for clarification on what it was or details of the policy itself.

Conversely, among those who seemed to understand decriminalization, most participants knowledge was based on their experiences. Some participants had observed a reduction in criminal penalties and policing in BC already. Here, they focused on the removal of criminal penalties that comes with decriminalization, including de facto decriminalization which according to many was already in place:

*“I thought decriminalization was already in effect…. that’s why they’re always just throwing away my stuff, so I thought it was already in effect personally, I didn’t realize it wasn’t until January… [police] will tell you [to] ‘keep it under wraps’… times that I had those interactions with them, was them not ever, like, really mentioning it, that I had those substances.”* (PWUD-01-12)

Decriminalization was about reducing or removing arrest for simple possession – something they witnessed in BC prior to the policy change – thus, underscoring the importance of personal experience shaping their knowledge of what decriminalization meant. This perspective was reinforced by participant reports of recent arrests: we found no reports of being detained or charged for simple possession in the past year among the qualitative sample and only 12% of people with recent police interaction in the quantitative sample identified being arrested for having drugs in the preceding three months.

### Conflating decriminalization with other drug policies

Participants often conflated or confused drug decriminalization with other laws and policy frameworks, highlighting a misunderstanding of what decriminalization was. Many participants used the term or idea of decriminalization interchangeably with ‘safer supply’ or regulation and legalization, often implying that they were the same policy framework: “…*if we get it decrimmed that means that maybe we can open a [safer supply] site up and have more people…We need a lot more of those places and that’s probably what’s going to happen.”* (PWUD-01–36). *“I have [heard of decriminalization]…like with the SAFER [safer supply program] program.”* (PWUD-01–34). Other participants confidently explained their assumption that decriminalization introduced a market where they could legally purchase drugs: “*Some people are confused with that [decriminalization], like: “it’s legal, it’s legal!*”” (PWUD-01–12). Participants also reported that such confusion or conflation was common amongst other PWUD in their community.

### The importance of understanding drug policy

Knowledge about the decriminalization model in BC was a key concern for PWUD in terms of their rights and empowerment. Several participants expressed fear around the perceived lack of knowledge that PWUD had surrounding BCs model of decriminalization and the disadvantage that this placed on them.

*“I didn’t really know anything…I think it’s pretty important that they know all of what you said. Like, if they’re just coming out of jail or if they got court orders or anything like that this decriminalization isn’t going to work in certain cases like that”* (PWUD-01-14)

Here, there is a sense that PWUD don’t trust that they still won’t be criminalized when the chance presents itself.

Such unknowns left PWUD feeling vulnerable to ongoing criminalization or police harassment. Participants were worried that poor understanding of the decriminalization policy would limit its effectiveness or benefits, as it may make them vulnerable to policing. Related to this vulnerability was a fear that police may negate the legal protections it affords if PWUD do not know their rights under BC’s decriminalization policy. Another person said:

*“If they’ve [PWUD] never heard of decriminalization and they’ve never heard that there’s a threshold, they [police officers] can do whatever they want to…I think [police officers] [are going to] be on people more because now they’re going to think, oh, if in fact we are informed about decriminalization, then people are…gonna not know about the threshold… trying to catch people that are ignorant and ill-informed.”* (PWUD-01-03)

Lacking knowledge of the details of decriminalization were a point of disempowerment or vulnerability in police encounters. The details themselves were important; for participants, there were risks in *knowing* about decriminalization generally, but not knowing its bounds or details specifically. For them, the general idea of decriminalization could give PWUD a false sense of security: *“It’s [decriminalization is] going to be difficult for people to understand…they’re going to learn the hard way by getting arrested or whatever.”* (PWUD-01–18). Other people similarly expressed concerns that misinformation or misunderstanding could also leave people vulnerable to other abuses of authority or misuse of the law:

*“Well, they need to clarify… the fine points of it [decriminalization]. Because it might cause a lot of problems for people, thinking they’re ok and they’re not, right…a lot of people…think it’s, you know, a golden ticket -- and it’s not.”* (PWUD-02-11)

PWUD continued to mistrust the law and enforcement despite knowing about decriminalization, assuming that they would be caught up in the finer details and penalized when given the chance. Lack of knowledge about decriminalization was a point of vulnerability for them in this arrangement.

Similarly, conflating decriminalization with other policy models falsely produced high expectations that decriminalization in BC was a drug market intervention or that it granted greater protections than it offered:

Participant:* “It’s [decriminalization] a good thing… people will have access to cleaner dope, and we wouldn’t be losing as many friends.”*

Interviewer:* “You mentioned having access to cleaner dope. How do you think that’ll come about with decriminalization?”*

Participant: *“Because it would be monitored…So, it would all be tested, so there would be nothing that would potentially kill somebody, right?”* (PWUD-01-17)

Such expectations produced a false sense of security or safety that could potentially introduce greater risks for PWUD, both in terms of criminalization and drug toxicity.

### Knowledge about decriminalization: what do people know about the details of decimalization?

In both the survey and interviews, we assessed PWUDs’ knowledge about the details of decriminalization through questions about specific elements of the exemption, that is, where/when decriminalization applies. People reported: *“I’ve heard the basic framework of it [decriminalization]. I don’t know the details” (*PWUD-02–11) – which is also reflected in the quantitative findings. Details included the parameters of BC’s decriminalization model, its limits, and the legal protections it afforded to PWUD. Specific elements examined in the quantitative and qualitative arms included the substances covered by the exemption, threshold quantity, drug seizures, and trafficking circumstances. Quantitative and qualitative outcomes of knowledge of exemption details were largely similar across demographic stratifications.

### Knowledge about the substances included

Among survey participants who were aware of decriminalization in BC (*n* = 254), 29% accurately identified all five drugs included. Accurate knowledge of the individual drugs included in decriminalization varied; methamphetamine was the highest (60%), followed by opioids (59%), powder cocaine (57%), crack cocaine (54%). A sub-analysis showed greater knowledge of the drugs included in decriminalization if the participant had used that drug in the past 30 days, particularly among people who use stimulants and/or opioids. People who used methamphetamine, powder cocaine, and opioids were significantly more likely to know whether the drug they used was included in decriminalization (Table 4). In contrast, only 25% (*n* = 20) of people who used MDMA were aware that this drug is included in decriminalization.

**Table 4:** Association between drug-specific awareness of BC’s decriminalization policy and use of that drug ^1^

		Awareness of decriminalization^3^				
	Users of drug	No or not sure		Yes		χ^2^_1_,*P*-value
		*n*	%	*n*	%	
MDMA^2^	No	221	66.2%	113	33.8%	0.32, 0.57
	Yes	15	75.0%	5	25.0%	
Powder cocaine	No	146	51.8%	136	48.2%	3.88, 0.049
	Yes	31	39.2%	48	60.8%	
Crack cocaine	No	129	54.2%	109	45.8%	1.11, 0.29
	Yes	62	48.4%	66	51.6%	
Methamphetamine	No	58	58.0%	42	42.0%	8.89, < 0.01
	Yes	110	40.0%	165	60.0%	
Heroin/Fentanyl	No	88	58.7%	62	41.3%	18.20, < 0.001
	Yes	80	36.2%	141	63.8%	

^1^Pearson’s Chi-squared test statistic and *p*-value

^2^Due to small cell size, Yates continuity correction applied to Chi-squared test

^3^Sample sizes in each chi do not include n = 404 due to missing responses for awareness or use of each drug

In the qualitative interviews, participants did not talk about the substances included at length but seemed confident in their knowledge about the substances included. Some participants recalled and listed them accurately with no hesitations. However, we did not specifically interrogate them more on this topic. Interestingly, excluded substances from decriminalization, including hallucinogens and benzodiazepines, were also not raised by or discussed among any participants, despite being present in the illegal market – potentially highlighting a knowledge gap or area for future investigation.

### Knowledge about the threshold amount

Approximately 45% (*n* = 98) of survey respondents who provided an estimate of the personal possession limit accurately estimated 2.5 g. While the median estimate was 2.5 g, the average estimate was 4.7 g, reflecting the participants who overestimated the allowable possession limit. However, it was unclear whether respondents believed the possession limit was above 2.5 g or whether they may have been providing information on an aspirational estimate.

In the qualitative interviews, many of the participants who had heard of decriminalization understood that BC was introducing a model with a threshold amount. This topic was talked about it at length by most participants, indicating a clear discourse that was present amongst their communities, although their knowledge about it varied. Overall, many participants accurately stated a threshold amount of 2.5 g, although a few overestimated 3.5–4.5 g or estimated under 1.5 g: “*Oh, it’s like probably not even half a gram*” (PWUD-01–20). Others did not know the amount: “*I know nothing about like the metric system and numbers are like a whole foreign language to me. So, I don’t really know*.” (PWUD-01–01). As well, it was mostly unclear whether PWUD understood that the 2.5 g threshold amount was cumulative of all drugs in possession, although some did offer this detail: “*The fact that it’s two and a half grams cumulative meaning that you can have a total amount of a combination of whatever’s being decriminalized, not two and a half grams of each. But a grand total of it.”* (PWUD-01–30).

### Knowledge about drug confiscation

In the survey, participants responded to true and false statements about drug confiscation and drug trafficking (Table 5 and 6). 41% (*n* = 104) of survey respondents aware of the upcoming exemption were either unsure or believed that police could still confiscate drugs under the incoming decriminalization policy. Approximately one in four respondents (22%) thought that police may seize drugs under the threshold amount, and one in five (19%) were unsure whether this was permitted. The belief that police could seize drugs under the threshold was significantly associated with participating at a site in Interior or Northern health authority compared to Fraser health and being from a medium sized community compared to a large community (Table 6). People aged 19–39 were 30% more likely to correctly answer that police cannot confiscate drugs under the threshold compared to those aged 40 and older (RR: 1.30 [95% CI: 1.09–1.55]). People with access to the internet were also 55% more likely to have knowledge of drug confiscation under decriminalization (RR: 1.55 [95% CI: 1.05–2.31]).

**Table 5:** True and False statements about the circumstances that BC’s decriminalization policy applies among survey participants aware of decriminalization (*n* = 254)

	**Police can take drugs if holding less than the allowable amount**		**People can be arrested for trafficking no matter how much drug they have on them**	
	***n***	**%**	**n**	**%**
**Respondents**	249	98.0%	247	97.2%
True	56	22.5%	136	55.1%
False	145	58.2%	63	25.5%
Not sure	48	19.3%	48	19.4%

^*^Correct answers: False *unless* in a location where the exemption does not apply or if subject to certain court conditions; True

**Table 6:** Associations between harm reduction client characteristics and correctly answering true or false questions about decriminalization among harm reduction clients aware of BC’s decriminalization policy

	**Accurately answered ‘false’ to: “police can take drugs if holding less than the allowable amount” (*n* = 249)**					**Accurately answered ‘true’ to: “people can be arrested for trafficking no matter how much drug they have on them” (*n* = 247)**				
	Risk Ratio	95% Confidence Interval		*P*-value	χ^2^_1_, *P*-value	Risk Ratio	95% Confidence Interval		*P*-value	χ^2^_1_,*P*-value
		Lower CI	Upper CI				Lower CI	Upper CI		
**Health Authority**					4.61, 0.33					3.90, 0.42
Fraser	Ref	Ref	Ref	Ref		Ref	Ref	Ref	Ref	
Interior	0.74	0.57	0.97	0.03		0.75	0.54	1.06	0.10	
Island	0.95	0.78	1.15	0.58		0.84	0.63	1.13	0.26	
Northern	0.72	0.53	0.99	0.04		0.96	0.63	1.46	0.86	
Vancouver Coastal	0.74	0.53	1.05	0.09		0.85	0.58	1.26	0.42	
**Community size**					11.38, < 0.01					2.23, 0.33
Large population	Ref	Ref	Ref	Ref		Ref	Ref	Ref	Ref	
Medium population	0.57	0.44	0.75	< 0.001		1.14	0.83	1.55	0.42	
Small population	0.85	0.69	1.05	0.13		1.16	0.83	1.63	0.39	
**Housing concerns past 6 months**					0.01, 0.91					0.64, 0.43
No	Ref	Ref	Ref	Ref		Ref	Ref	Ref	Ref	
Yes	1.12	0.93	1.35	0.24		0.98	0.79	1.22	0.86	
**Internet Access**					4.71, 0.03					1.42, 0.23
No	Ref	Ref	Ref	Ref		Ref	Ref	Ref	Ref	
Yes	1.55	1.05	2.31	0.03		1.28	0.88	1.85	0.20	
**Age group**					5.14, 0.02					2.05, 0.15
40 and older	Ref	Ref	Ref	Ref		Ref	Ref	Ref	Ref	
19–39	1.30	1.09	1.55	0.003		0.82	0.65	1.03	0.08	
**Sex/gender**					0.002, 0.96					1.27, 0.26
Cis man	Ref	Ref	Ref	Ref		Ref	Ref	Ref	Ref	
Cis woman	1.02	0.89	1.17	0.73		0.85	0.66	1.09	0.21	
**Opioid use past 3 days**					3.06, 0.08					0.12, 0.72
No	Ref	Ref	Ref	Ref		Ref	Ref	Ref	Ref	
Yes	1.09	0.92	1.28	0.31		1.27	0.99	1.62	0.06	
**Simulant use past 3 days**					1.48, 0.22					1.32, 0.25
No	Ref	Ref	Ref	Ref		Ref	Ref	Ref	Ref	
Yes	1.07	0.88	1.30	0.49		0.90	0.71	1.14	0.37	

^1^Pearson’s Chi-squared test statistic and *p*-value

In the interviews, similar variations in knowledge about drug confiscation were reported, although most were aware that it was a feature of the exemption. In introducing this topic to interview participants, most were quick to express skepticism that decriminalization would reduce drug confiscation at all. This skepticism was based on a sense of deep distrust towards the law and officer. They believed that police officers would continue to confiscate and/or destroy drugs regardless of the policy: “*As far as the decrim goes, what do they do? So, they’ll still probably take your drugs, right? They just won’t charge you.”* (PWUD-01–09). On this topic, many reported that drug confiscation was a regular and frequent occurrence. Their distrust was reinforced by the belief that decriminalization lacked any measures or plans to hold police officers accountable or monitor their practices. In combination, PWUDs’ distrust, beliefs and experiences overlayed their judgement or confidence in decriminalization and confiscation altogether.

### Knowledge about drug trafficking under decriminalization

Under Canada’s possession for the purposes of trafficking laws, when evidence of trafficking is present (e.g., scale, cell phone, baggies) police officers may have just cause for arrest and pursuing charges regardless of the amount in possession. Over half of survey respondents (*n* = 136, 55%) (Table 5) knew that people could still be arrested and charged for drug trafficking following decriminalization.

Knowledge and understanding of drug trafficking laws was talked about at length in the qualitative interviews; how drug trafficking charges would be handled by police officers and the courts was one of the most confusing details of decriminalization for participants.

“If you just had one big bag of dope, even if you had-- it’s the scales thing that makes it to the trafficking, I think. I’m not sure. I don’t know the current rules around what the-- like, how much you’re allowed to have before it’s a [trafficking] charge.” [PWUD-01-32]

Some believed even drug trafficking offences under 2.5g were covered, whereas others assumed the market was decriminalized (or legalized) altogether – again, reflecting conflation with other legal models. Participants were especially unclear on how drug trafficking and possession charges would be delineated from each other – a piece of information that was especially important for PWUD who felt vulnerable to policing otherwise.

*“It’s [decriminalization is] a welcome relief for a lot of people, but like I said… it’s causing a bit of confusion… I don’t think people understand, you know, like, that you can still get popped for trafficking. So, like, for them, if it’s [drugs are] all busted up in little packages they’ll consider that as trafficking, right. You know, there is still some grey areas*."

The possibility of arrest or charges for drug trafficking still left uncertainty in police interactions where, in lieu of specific details and parameters for drug trafficking, they were not confident in the outcomes.

### Sources of information: How did people become aware of decriminalization or get their information?

Survey respondents indicated where they received information about BC’s incoming decriminalization policy (Table 7). Among survey participants aware of decriminalization (*n* = 248), nearly 98% of respondents indicated their information source about decriminalization. The most common source was community organizations that provide support and services for PWUD such as harm reduction sites, overdose prevention sites, and supervised consumption sites (58%), indicating that this avenue may play an important role in ongoing education and support for PWUD in understanding their rights under decriminalization. Many respondents also knew about decriminalization from friends (41%), on the news/media (29%), their health care provider (26%), and/or from a drug user group (25%).

**Table 7:** Sources of information about BC’s decriminalization policy among harm reduction clients (*n* = 404)

			**Aware of decriminalization**			
	**Overall (*n* = 404)**		**Yes (*n* = 254)**		**No (*n* = 150)**	
	***n***	**%**	***n***	**%**	***n***	**%**
Checked any	393	97.3%	248	97.6%	145	96.7%
Harm reduction site / OPS / SCS / community organization	217	55.2%	144	58.1%	73	50.3%
Health care provider	89	22.6%	64	25.8%	25	17.2%
On social media (Facebook / Twitter / TikTok, etc.)	80	20.4%	59	23.8%	21	14.5%
On the news/media	98	24.9%	72	29.0%	26	17.9%
Friend	140	35.6%	101	40.7%	39	26.9%
Drug user group	85	21.6%	61	24.6%	24	16.6%
Dealer	59	15.0%	40	16.1%	19	13.1%
Posters on the street	44	11.2%	34	13.7%	10	6.9%
This survey	159	40.5%	80	32.3%	79	54.5%
Other	32	8.1%	18	7.3%	14	9.7%

Of survey respondents, 40% indicated that they became aware of decriminalization from the survey itself. Learning about decriminalization from the survey was higher for people not previously aware of decriminalization (50%) than those previously aware (32%). It was unclear whether respondents endorsed sources of information they had used, preferred sources of information, or whether some may have received the decriminalization handout before they completed the survey.

Similar to survey respondents, qualitative participants indicated that the interview itself was the first time they became aware of decriminalization: *“No one has ever explained it like that before. Wow.”* (PWUD-01–04)*.* It was clear from the qualitative interviews that information about decriminalization, both in terms of availability and depth of information, was scarce or lacking at the time of data collection – immediately before the inception of decriminalization. Some participants reported that they had received some but minimal information through word-of-mouth from harm reduction sites/community organization, peer-based networks, and friends. Some participants thought that information about decriminalization was largely limited to social media or news outlets – avenues that made some PWUD aware but not overly knowledgeable about the policy. As well, limited information through these outlets was an issue as news and access to the internet was not available for all PWUD: *“No, I didn’t hear anything. I don’t watch the news.”* (PWUD-01–04). For PWUD, this lack of information sharing about decriminalization was concerning in terms of equity: *“What worries me is not everybody knows and a lot of people that don’t know don’t have a way to really find out, they don’t have internet or whatever.”* (PWUD-01–20). In other words, participants questioned who decriminalization benefited if it was not known among equity-deserving groups. Examples provided by participants of such groups included people who are unhoused or precariously housed or who had visual impairments.

From PWUDs’ reflections about BC’s decriminalization, there was a clear desire to receive more information and see greater education efforts from the government, harm reduction organization and other advocacy groups. Participants suggested several recommendations for potential avenues for information sharing, including ‘know-your-rights’ trainings, but mainly participants emphasized that information be shared through multiple avenues to increase the reach to the community:

*“It should be on TV, commercials, everybody should know about it… more so that people know, if they don’t know, it’s still going to be the same shit. Even if it changes tomorrow, it will still be the same pretty much. We need to make people more aware.”* (PWUD-01-02).

PWUD also emphasized information sharing through peer workers and harm reduction organizations to reach their communities: “*There’s lots of peers out working, you know…people who are out there doing outreach”* (PWUD-02–11). For them, peer workers who had strong relationships to the community were well positioned to engage in accurate knowledge sharing. Several participants identified as peer workers and talked about how they could leverage and share information: “*As a peer worker I know my rights”* (PWUD-01–03). In addition to accuracy and being well-connected, they talked about peer workers having first-hand experience with the legal system that they believed was valued by other PWUD.

## Discussion

In this multi-methods study, we examined PWUDs’ awareness and knowledge of decriminalization immediately prior to its implementation in attempts to understand knowledge gaps. We found that over half of PWUD who participated in the survey were aware of the incoming legal change and through both the qualitative and quantitative findings, that there were knowledge gaps in PWUDs’ understanding and knowledge about it. PWUD often did not understand the parameters of the decriminalization model, including the drugs, amounts, and circumstances in which it applies. Findings identify several groups that may be vulnerable to ongoing criminalization due to their lack of awareness and/or understanding, including people without stable housing, people without access to the internet, who are disconnected from services, and other equity-deserving groups. These findings are perhaps not surprising given PWUDs’ qualitative reports of a lack of education or knowledge sharing about it to the community. To ensure that the benefits of decriminalization are realized following its implementation, there are ample opportunities to bolster knowledge sharing to PWUD about BC’s decriminalization model. As discussed below, participants offered several recommendations for increasing awareness and understanding of decriminalization in the community.

The finding that approximately two-thirds of PWUD in our study were aware of decriminalization is slightly elevated compared with other studies that have examined PWUDs’ awareness of drug policy reforms. A study conducted in BC on awareness of Canada’s Good Samaritan Drug Overdose Act in 2020 found that just over half of PWUD were aware of the reform [60], while other jurisdictions report lower awareness of local drug-related good Samaritan laws [50, 52, 61]. Awareness of other drug policies among PWUD globally are considerably lower. For example, among a sample of 737 PWUD in Mexico, knowledge of the country’s decriminalization regime was only 11% [53]. We expect that as decriminalization in BC is implemented, awareness may increase; however, it is unclear how knowledge and information about the model may evolve. Future iterations of the Harm Reduction Client Survey and qualitative research by our teams are planned to assess ongoing awareness, understanding, and knowledge of decriminalization post-implementation.

While over half of participants were aware of decriminalization in BC, understanding the model and details of decriminalization among PWUD was highly variable. The qualitative findings provide nuance as to where discrepancies exist. Interview participants often conflated decriminalization with other legal frameworks (i.e., regulation or ‘safer supply’). This finding reflects other studies showing that PWUD, police officers, and the public do not understand what is meant by the term ‘decriminalization’ [62–64]. As noted in previous work, the details of a decriminalization model can be complex and therefore obscure [65]. Being aware of decriminalization, yet misunderstanding its benefits and limits, can produce a false sense of security that can unknowingly result in penalties and/or police attention. Findings underscore the need to clearly define and articulate this legal parameters among target audiences, including the goal or what the policy is intended for.

Concerns for the impact of knowledge disparities has been echoed in other studies where legal protections from drug policies were limited. For instance, the benefits of Canada’s Good Samaritan Drug Overdose Act were inflated amongst PWUD who believed that warrants and drug trafficking were covered under the legislation [25, 60]. Ample research demonstrates the impact of misunderstanding drug-related good Samaritan laws include ongoing hesitation to calling 9–1-1 in the event of an overdose, and an ongoing fear and distrust towards police, the legal system, and government in general [39, 60, 66].

Echoed throughout our findings is evidence of PWUDs’ mistrust towards police officers and the law that is based in a long history of conflict. In previous studies, PWUD have reported violence, abuses of power, misconduct, discrimination, and a lack of procedural justice contribute to this mistrust [30, 38, 67–71]. These factors are often linked to intersecting structural vulnerabilities that position some PWUD at greater risk of police contact and negative outcomes [69, 70, 72]. In their report on decriminalization in Portugal, the International Network of People who Use Drugs (INPUD) note: “…full decriminalisation requires a meticulous dismantling of the structures, policies, and practices of prohibition and its associated harms, including the power dynamics that typically govern the relationship between police and people who use drugs” [73]. It is therefore important to ensure decriminalization in BC is effectively implemented in a way that promotes structural change whereby policing is decoupled from the lives of PWUD and healthcare.

Conversely, participants in our study suggested that increasing knowledge about their rights under decriminalization has the potential to empower PWUD in police interactions and promote the benefits of policy change, including de-stigmatization and a sense of social inclusion. In addition to structural changes that are needed, additional knowledge sharing with police officers, training, and accountability measures that promote procedural justice may need to be in place and communicated to PWUD to promote trust and legitimacy of the policy itself. Effective health communication strategies have been shown to be a key element of health policy implementation. The strategies suggested by participants in the current study, including leveraging peer networks and technology, are shown to be effective in the health communication literature. Such strategies could be effective in addressing the clear gaps in knowledge among PWUD in BC. Knowledge of the policy considerably varied by region, such as in rural and remote areas where misconceptions of the policy existed (e.g. awareness was the lowest in the North and more participants in the Northern Health Authority believed that police officers could seize drugs). This finding aligns with health communication findings across a range of health issues showing that reaching groups in rural regions is significantly more challenging than urban populations [74, 75], due to infrastructure, the cultural environment, information sources, and fiscal costs [75–79]. Innovative health communication interventions, such as the use of technology and social media, could promote health literacy in rural communities, although connectivity and accessibility may need to be addressed [75, 80, 81]. Groups from rural areas should be engaged in the design and delivery of such campaigns as they understand the rural context and culture in which people live [74, 79]. Other studies in BC show that communication about drug alerts occurred mainly through friends or peers in rural communities [82] – therefore, networks of PWUD may be important to connect with and distribute health information through.

Based on our findings, knowledge about BC’s decriminalization policy was limited to news media sources and word-of-mouth, ultimately placing the burden on PWUD to seek out and comprehend information about it. “A comprehensive public education and communications plan” was listed as a key commitment and requirement of the exemption itself [83]. Despite this commitment, there was limited communication about the amendment from the Government of BC beyond information listed on its own website [13], despite “a comprehensive public education and communications plan” being listed as a key commitment and requirement of the exemption itself. Information sharing should consider barriers that PWUD may face to accessing online information, including limited cell phone ownership [84]. As participants in our study noted, PWUD are a diverse population with differing knowledge sharing needs. Participants strongly suggested that multiple and diverse avenues for information sharing are needed, including through social media, harm reduction sites, and peer networks. To ensure that the exemption can equitably reduce criminalization, education efforts should be targeted to people with lower awareness of the policy and its components, including women, younger people, people without access to the internet, people with visual impairments, and people who live in rural and remote communities. Previous research has shown that particular forms of knowledge sharing work well with this community, including from peers and other PWUD [85, 86]. Since collecting data in our study, some community groups have acted in attempts to increase knowledge and awareness among PWUD, including Pivot Legal Society know your rights training and information cards [87].

These data were collected as a pre-implementation baseline. Results may have been different in the period after data collection but before implementation (November 2022-January 2023). As well, it may be that PWUD who access harm reduction organizations/services are informed more than others – particularly because we found harm reduction sites were a main source of information for them. Research with more hidden, precarious, or less connected PWUD may produce different findings and is an area of future research.

## Conclusion

Decriminalization in BC is a historic policy change and pilot reform that will be in place for three years, from January 2023–2026. The success of this reform will be judged on multiple outcomes, including reducing criminal penalties, stigma, and increasing access to health and harm reduction services. These benefits of decriminalization may hinge on awareness, understanding, and knowledge of decriminalization among impacted groups. Findings from our pre-implementation multi-methods study suggests that these factors may be deficient among PWUD in BC – the very community that decriminalization is intended to have a positively impact. Considering that the success of decriminalization and its benefits may be undermined by poor awareness and knowledge of it, efforts to share information, increase understanding, and empower the community, are a key part of its implementation.

## Data Availability

The datasets generated and/or analysed during the current study are not publicly available under current ethics approvals and to promote confidentiality but may be available from the corresponding author on reasonable request.

## Abbreviations

BC: British Columbia
PWUD: People who use drugs
CI: Confidence Interval
RR: Risk Ratio
